# Editorial: Navigating challenges and innovations in antimicrobial resistance, environmental microbiology, and industrial solutions (ARTEMIS)

**DOI:** 10.3389/fmicb.2025.1677866

**Published:** 2025-08-22

**Authors:** Arpit Shukla, Dweipayan Goswami, Chaitanya Kumar Jha

**Affiliations:** ^1^Cancer Research Centre, University College Cork, Cork City, Ireland; ^2^School of Biotechnology and Bioengineering, Institute of Advanced Research, Gandhinagar, Gujarat, India; ^3^Department of Microbiology and Biotechnology, University School of Sciences, Gujarat University, Ahmedabad, Gujarat, India; ^4^Microbiology Department, Gujarat Arts and Science College, Gujarat University, Ahmedabad, Gujarat, India

**Keywords:** antimicrobial resistance, drug resistance, microbial ecology, drug discovery, quorum sensing, medical microbiology, bioinformatics, industrial microbiology

The silent pandemic of Antimicrobial Resistance (AMR) continues to escalate, posing one of the most significant threats to global health, food security, and development in the 21st century. The complex web of factors driving its emergence and spread transcends clinical settings, deeply embedding itself within our environment and industrial practices. It is from this understanding that our Research Topic, “*Navigating Challenges and Innovations in Antimicrobial Resistance: Environmental Microbiology, and Industrial Solutions*,” was born.

This Research Topic is proud to be organized in collaboration with the 2-day National Conference on ARTEMIS 2024, held on the 21st and 22nd of March, 2024, at the Institute of Advanced Research in Gandhinagar, India. This pivotal event, sponsored by the Gujarat State Biotechnology Mission (GSBTM), the Science and Engineering Research Board (SERB), and Frontiers, brought together the next generation of scientists to address this global challenge. The conference was a resounding success, hosting 140 participants and featuring vibrant scientific exchange through 27 oral and 46 poster presentations from postgraduate and doctoral students. This Research Topic of articles was conceived to create a lasting, peer-reviewed platform for the ideas and research discussed, uniting researchers to explore the intersections of these critical fields and fostering a holistic approach to understanding and combating AMR. The diverse and innovative articles presented here are a testament to that collaborative vision.

The contributions to this Research Topic highlight three interconnected pillars: the direct challenge of AMR, the crucial role of environmental microbiology, and the development of sustainable industrial and agricultural solutions.

Several articles tackle the challenge of AMR head-on, focusing on improved detection and novel therapeutic strategies. The development of rapid and accurate diagnostics is paramount to effective surveillance and treatment. Addressing this, the work by Zhu et al. provides a valuable new tool with their evaluation of a two-dimensional PCR method for the rapid detection of hypervirulent *Klebsiella pneumoniae*, a notoriously difficult pathogen. Complementing this, Liang et al. developed three simple and cost-effective assays to detect the activity of the AAC(6′)-Ib-cr enzyme, which confers resistance to multiple antibiotics, offering a practical solution for resource-limited settings. Beyond detection, the search for alternatives to conventional antibiotics is urgent. Wang et al. present a promising approach by displaying a prophage lysin on the surface of *Bacillus subtilis* spores, creating a potent antibacterial agent against the swine pathogen *Streptococcus suis*. In a similar vein, Liu et al. explore the natural world for solutions, providing a preliminary but encouraging look into the antimicrobial and antioxidant potential of *Magnolia* essential oil, a potential source for novel bioactive compounds.

The environment is a critical reservoir and conduit for the spread of resistance genes and resistant organisms. Several of our authors have focused on harnessing the power of microorganisms to remediate and protect our ecosystems. Recognizing that contaminated waterways are hotspots for AMR, Patel et al. demonstrate an innovative solution using a synergistic system of plants and bacteria in floating treatment beds to efficiently remove emerging contaminants from polluted river water. Directly addressing pharmaceutical pollution, Yang et al. isolated and characterized bacteria capable of efficiently degrading the antibiotic neomycin, offering a bioremediation strategy for contaminated wastewater and soil. The impact of industrial pollution is tackled by Zheng et al., who characterized a bacterium from activated sludge capable of degrading N,N-dimethylformamide, a widely used industrial solvent, showcasing the power of microbes in waste treatment.

Finally, this Research Topic underscores the immense potential of microbial solutions in industrial and agricultural contexts, which are key to a sustainable future. In agriculture, Du et al. show how thermophilic microbial agents can accelerate the composting of pig manure and spent mushroom substrate, transforming agricultural waste into a valuable resource. Shifting from waste management to crop protection, Kamath et al. explore the use of siderophores produced by bacteria to defend mung bean plants against fungal disease, presenting a biological alternative to chemical fungicides that can inadvertently drive resistance.

Together, these articles paint a hopeful picture. They showcase a dynamic research landscape where innovative diagnostics, nature-inspired antimicrobials, and powerful bioremediation strategies are being developed. The findings collected here reinforce the necessity of a “One Health” perspective, where the intricate links between human, animal, and environmental health are not just acknowledged but are central to our research endeavors. We extend our sincere gratitude to all the authors who contributed their valuable research and to the reviewers whose diligent efforts upheld the quality of this work. It is our hope that this Research Topic, born from the energy and intellect of the ARTEMIS 2024 conference, will stimulate further discussion, collaboration, and innovation, moving us closer to a world where the threat of antimicrobial resistance is effectively contained.

